# MHD Flow of the Micropolar Fluid between Eccentrically Rotating Disks

**DOI:** 10.1155/2014/317075

**Published:** 2014-09-03

**Authors:** Neetu Srivastava

**Affiliations:** Department of Mathematics, Amrita Vishwa Vidyapeetham (Deemed University), Bangalore 560035, India

## Abstract

This analytical investigation examines the magnetohydrodynamic flow problem of an incompressible micropolar fluid between the two eccentrically placed disks. Employing suitable transformations, the flow governing partial differential equations is reduced to ordinary differential equations. An exact solution representing the different flow characteristic of micropolar fluid has been derived by solving the ordinary differential equations. Analysis of the flow characteristics of the micropolar fluid has been done graphically by varying the Reynolds number and the Hartmann number. This analysis has been carried out for the weak and strong interactions.

## 1. Introduction

Non-Newtonian fluids are the most widely occurring fluids in industry and engineering and its governing equations are highly nonlinear as compared to the Navier-Stokes equation. Non-Newtonian fluids are complex in nature because their rheological properties are involved as “parameters” in the constitutive equation. Such fluids cannot be characterized by the single expression because of its diverse characteristics. In spite of its complex nature, several researchers worked for this fluid and studied the non-Newtonian fluid flow characteristics on the various geometrical configurations. Eringen [1–3] presented the theory of non-Newtonian fluid which is completely different from the other non-Newtonian fluids because of its microscopic properties like microrotational and the rotational inertia. Such fluids are called micropolar fluids. Main advantage of using micropolar fluid model to study the fluid flow in comparison with other classes of non-Newtonian fluids is that it takes care of the rotation of fluid particles by means of an independent kinematic vector called the microrotation vector. These are very important for the analysis of flow characteristics of micropolar fluid like colloidal suspension, liquid crystals, fluids with additives, suspension solutions, animal blood, inkjet and printing, microfluidics, geological flows in the earth mantle, homodynamic liquid crystals, additive suspensions, and many others. It is difficult to obtain the solution of the governing equations for the micropolar fluid as they are highly nonlinear in nature. A lot of work [1, 4–13] has been carried out regarding the analytical solution for the flow of non-Newtonian fluids in cases where the involved equations have been linearized and in cases where partial differential equations have been reduced to ordinary differential equations.

MHD boundary layer problems are of great importance in understanding a variety of geophysical, astrophysical, and engineering phenomena like the geophysical phenomena that occur at the core-mantle interface of the earth. The flow of a viscous incompressible fluid between two parallel plates rotating noncoaxially but with the same angular velocity was studied by Berker [14]. Coirier [15] analysed the flow due to a disk and a fluid at infinity which is rotating noncoaxially at slightly different angular velocity. Abbott and Walters [16] studied the hydrodynamic flow between two disks, rotating with the same angular velocity about noncoincident axes. Mohanty [17] investigated the magnetohydrodynamic flow between two disks, rotating with the same angular velocity about two different axes neglecting the induced magnetic field. An extension to this problem has been done by Rao and Kasiviswanathan [18]. Other extensions of this type of flow to an Oldroyd-B fluid were studied by Rajagopal [19] and in case of electrically conducting Oldroyd-B fluid by Ersoy [20]. Erdogan [21] studied the unsteady hydrodynamic viscous flow between eccentric rotating disks. Rao and Kasiviswanathan [22] considered the flow of an incompressible viscous fluid between two eccentric rotating disks for unsteady cases. Lai et al. [23] discussed the three-dimensional flow between two parallel plates which are rotating about a common axis or about distinct axes. Knight [24] investigated the inertia effects of the non-Newtonian flow between eccentric disks rotating at different speeds. Guria et al. [25–28] have studied the noncoaxial rotations of two porous disks or the rotations of porous disk and a fluid at infinity under different environments.

Recently, Guria et al. [27] have discussed the MHD squeezing flow of a micropolar fluid between parallel disks. They derived similarity solutions by homotopy analysis method and compared them with the solutions obtained by homotopy perturbation method. Guria et al. [28] have discussed an unsteady MHD flow between two eccentric rotating disks. They discussed the solutions obtained by applying the integral transforms technique. Shercliff et al. [29] have discussed an axisymmetric magnetohydrodynamic flow of micropolar fluid between unsteady stretching surfaces. They discussed the solutions obtained by applying the homotopy analysis method.

Hence, in the view of above literature survey, the objective of the present paper is to study the magnetohydrodynamic flow of the micropolar fluid between eccentrically placed rotating disks. This study has been carried out to show the effects of the Hartmann number and micropolar parameter on the velocity and microrotation parameter.

## 2. Mathematical Formulation and Its Solution

Capriz [30] and Mariano [31] defined multifield continua by including some additional measures of strain and stress in the constitutive relation of a continuous model (i.e., by including scaling effect). These continua are the continua with any kind of local microstructure and represent an extension of the continua with kinematical microstructures defined in classical work. The classical continuum mechanics are augmented with additional equations that account for conservation of microinertia moments and balance of first stress moments that arises due to consideration of the microstructure in a material. When the scaling effect is negligible, the classical and the micropolar fluid model coincide. This micropolar model and its field equation is introduced by Chen et al. [13] and Eringen [1, 2] are

(1) ∂ρ∂t+div⁡(ρv−)=0,ρdv−dt=ρf−∇p+κ∇−×N−−(μ+κ)∇−×∇−×V−+(λ+2μ+κ)∇(div⁡V−),ρJdN−dt=ρI−2κN−+κ∇−×N−−γ∇−×∇−×N−+(α+β+γ)∇(div⁡N−),

where *ρ* is the density, $\overset{-}{V}$ is the velocity field, $\overset{-}{N}$ is the microrotation field, $\overset{-}{J}$ is the gyration parameter, *f* is the body forces per unit mass, *I* is the microrotation driving forces per unit mass, *p* is the hydrostatical pressure, *μ* is the classical viscosity coefficient, *κ* and *λ* are the vortex viscosity coefficients, and *α*, *β*, and *γ* are gyroviscosity coefficients satisfying the following inequalities:

(2) 3α+β+γ≥0,  2μ+κ≥0,  3λ+2μ+κ≥0,mmmmmmmmiiiiiiimiimimγ≥|β|,  κ≥0,  γ≥0.

The constitutive equations for the stress tensor ${\overset{-}{\tau }}_{ij}$ and couple stress tensor  ${\overset{-}{m}}_{ij}$ are expressed by following equations, respectively, as:

(3) τ−ij=(−p+div⁡V−)δij+(2μ+κ)dij+κϵijm(ξ−N),m−ij=(αdiv⁡V−)δij+βξi,j+γξj,i,

where *d*_*ij*_ denote the rate of strain components and *N* and *ξ* are the components of microrotation vector and vorticity vector, respectively, and *δ*_*ij*_ is the Kronecker delta.

**Figure 1:**
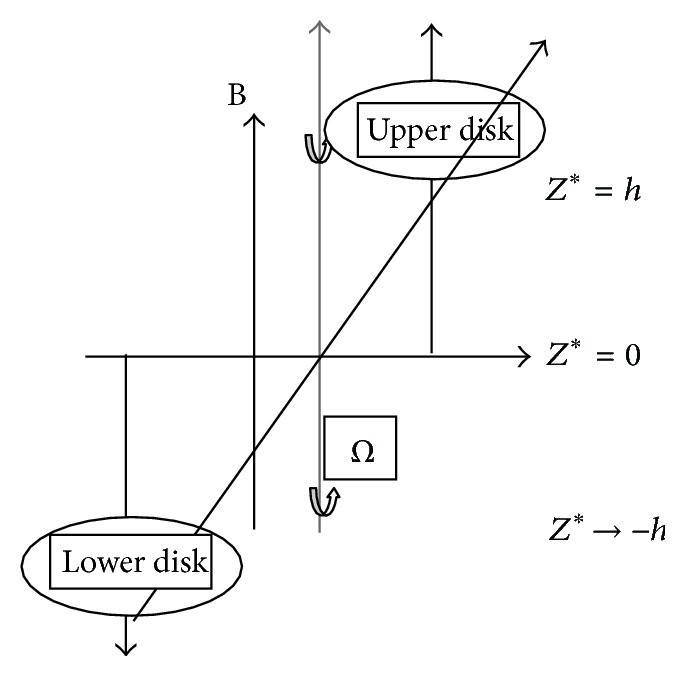
Schematic of the problem.

Consider the viscous incompressible fluid flowing through the space between the noncoaxial disks. In a Cartesian coordinate system, let us consider a rotation of eccentrically placed disks in *XY* plane rotating at a constant rate of *Ω* with *z* axis perpendicular to the plane of disks.  Figure 1 describes the geometry of the problem showing that the upper disk is placed at (0, *l*, *h*) and the lower disk is placed at (0, −*l*, −*h*). A uniform magnetic field of the strength Bo is applied in the normal direction to the disk. We assume that the induced magnetic field is negligible in comparison to the applied magnetic field.

The above equations ((1)–(3)) can be rewritten for an incompressible steady micropolar fluid in the presence of MHD (neglecting the body forces and couple terms) through the space between two noncoaxial disks and takes the form as follows:

(4) $$\begin{array}{c} \overset{-}{\nabla }\cdot \overset{-}{V}=0, \end{array}$$

(5) $$\begin{array}{c} 0=-\nabla p+\left(\mu +\kappa \right)\nabla^{2}\overset{-}{V}+\kappa \overset{-}{\nabla }\times \overset{-}{N}+\overset{-}{J}\times \overset{-}{B}, \end{array}$$

(6) 0=(α+β+γ)∇−(∇−·N−)−γ∇−×(∇−×N−)+κ∇−×N−−2κN−,

(7) $$\begin{array}{c} \overset{-}{J}=\sigma \left(\overset{-}{E}+\overset{-}{V}\times \overset{-}{B}\right), \end{array}$$

(8) $$\begin{array}{c} \overset{-}{\nabla }\times \overset{-}{E}=0, \end{array}$$

where $\overset{-}{V}$, $\overset{-}{N}$, and $\overset{-}{B}$ represent the velocity vector, micromotion vector, and the magnetic field vector, respectively, $\overset{-}{E}$ is the electric field vector, $\overset{-}{J}$ is the current density, *j* is the microinertia per unit mass, *Ω* is the angular velocity, and *ρ* is the fluid density for the considered problem. Furthermore, *μ*, *κ*,  *α*, *β*, and *γ* satisfy the following conditions:

(9) $$\begin{array}{c} 2\mu +\kappa \geq 0,\;\kappa \geq 0,\\ 3\alpha +\beta +\gamma \geq 0,\;\gamma \geq \left|\beta \right|. \end{array}$$

We will assume that the magnetic Reynolds number for the flow is small so that the induced magnetic field can be neglected. This assumption is justified since the magnetic Reynolds number is generally small for partially ionized gases. If (*B*_*x*_, *B*_*y*_, *B*_*z*_) are the components of the magnetic field vector $\overset{-}{B}$, the solenoidal relation $\nabla \cdot \overset{-}{B}=0$ gives *B*_*z*_ = constant = *B*_0_, everywhere in the fluid. Further, the equation of the conservation of the charge $\nabla \cdot \overset{-}{j}=0$ gives *j*_*z*_ = constant, where (*j*_*x*_, *j*_*y*_, *j*_*z*_) are the components of the current density $\overset{-}{j}$. This constant is zero since *j*_*z*_ = constant at the disk which is electrically nonconducting. Using (7), we obtain

(10) jx=σ(Ex+vB0),  jy=σ(Ey−uB0),jz=σ(Ez).

Since the induced magnetic field is neglected, Maxwell's equation (8) and (10) gives ∂*E*_*x*_/∂*z* = 0 and ∂*E*_*y*_/∂*z* = 0. This implies that *E*_*x*_ and *E*_*y*_ are independent of *z* everywhere in the flow. Let *u*, *v*, and *w* are the components of the velocity $\overset{-}{V}$ and *N*_1_, *N*_2_, and *N*_3_ are the components of the micropolar rotation vector $\overset{-}{N}$; then the equation of motion for the steady flow can be written as

(11) u∂u∂x+v∂u∂y=−1ρ∇p  +(ν+κρ)[∂2u−∂x2+∂2u−∂y2]+κρ∂N−∂x−σB02u−ρ,u∂v∂x+v∂v∂y=−1ρ∇p  +(ν+κρ)[∂2v−∂x2+∂2v−∂y2]+κρ∂N−∂y−σB02v−ρ.

Angular momentum equation can be written as

(12) u∂N−∂x+v∂N−∂y=(γρj)[∂2N−∂x2+∂2N−∂y2]−κρj(2N−−∂v∂x+∂u∂y),

where *j* = *νh* ^2^ and *γ* = (*μ* + (*κ*/2))*j* in which *μ* is the dynamic viscosity, *κ* is vortex viscosity, and *η* = *z*/*h*.

To nondimensionalize the governing equation, the above geometry of the problem suggests taking the form for the velocity and the microrotation vector as

(13) $$\begin{array}{c} u=-\Omega y+f\left(z\right),\;\;v=\Omega x+g\left(z\right),\\ w=0,\;\;N=hG\left(z\right). \end{array}$$

The boundary conditions for the problem can take the following form:

(14) u=−Ω(y−l),  v=Ωx,  w=0,  G=−n∂u∂ziiiiiiiiiiiiiiiimiiat  z=h,u=−Ω(y+l),  v=Ωx, w=0,  G=−n∂u∂zmiiiiiiiiiiii.mat  z=−h,

where *n* is the constant defined by 0 ≤ *n* ≤ 1. Here, *n* = 0 depicts the situation when microelements at the surface of disks are unable to rotate. This is also known as strong concentration of the microelements, whereas *n* = 0.5 depicts the situation of weak concentration of microelement at the surface of disks. For  *n* = 1, one can have turbulent boundary layer flow. Invoking the above transformation (13) in (11) and (12), we have

(15)  0=−1ρ∂p∂x+(ν+κρ)f′′−σB02ρ(f−Ωy)+(κρ)∂N−∂x+Ω(g+Ωx),

(16)  0=−1ρ∂p∂y+(ν+κρ)g′′−σB02ρ(g−Ωx)+(κρ)∂N−∂y+Ω(f−Ωy),

(17)  0=(νΩρjh)G′′−(2Kρj)(hG−Ω).

And eliminating the pressure from (15) and (16), we can have an ordinary differential equation of the form

(18) (1+K)F′′(η)+(M2+iRe2)F(η)=0,G′′(η)−2KG(η)=−2K,

where *F*/*Ωl* = (*f* + *ig*)/*Ωl*, the Hartmann number *M* ^2^ = *σB*_0_^2^ *h* ^2^/*ρυ*, the Reynolds number *Re* ^2^ = *Ωh* ^2^/*υ*, and the micropolar parameter *K* = *κ*/*μ*. The boundary conditions (14) take the following form:

(19) F=Ωl,  G=−nΩf′(1) at  η=1,F=−Ωl,  G=nΩf′(−1) at  η=−1.

Solving the above differential equations (15) and (16) with the boundary conditions (19), we can have a solution of the form

(20)  F(η)=C[1]esη+C[2]e−sη, G(η)=1+C[3]e2Kη+C[4]e−2Kη,

where

(21)  C[1]=Ωl[es+e−s][e2s−e−2s], C[2]=−Ωl[es+e−s][e2s−e−2s], C[3]=n[e−2Kf′(1)+e2Kf′(−1)]+[e−2K−e2K][1−e42K]−[nf′(1)+1]e2K, C[4]=−n[e−2Kf′(1)+e2Kf′(−1)]−[e−2K−e2K][e−22K−e22K].

The solution (16) can be rewritten as

(22) $$\begin{array}{c} \frac{F\left(\eta \right)}{\Omega l}=\frac{\sinh\left[\left(\eta +1\right)\left(\alpha +i\beta \right)\right]+\sinh\left[\left(\eta -1\right)\left(\alpha +i\beta \right)\right]}{\sinh\left[2\left(\alpha +i\beta \right)\right]}, \end{array}$$

where α=[(M2±M4+Re4)/2(1+K)]1/2, β=[(-M2±M4+Re4)/2(1+K)]1/2, and s=(M2+iRe2)/1+K.

For no microrotation, that is, for *n* = 0, the constants *C*_[3]_, *C*_[4]_ are

(23) C[3]=[e−2K−e2K][1−e42K]−[1]e2K,C[4]=−[e−2K−e2K][e−22K−e22K].

## 3. Results

**Figure 2:**
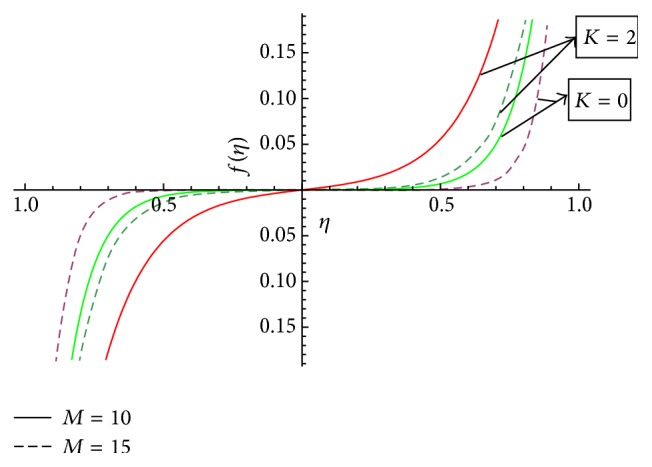
Variation of primary velocity *f*(*η*) with the Hartmann number (*M*) and micropolar parameter (*K*).

**Figure 3:**
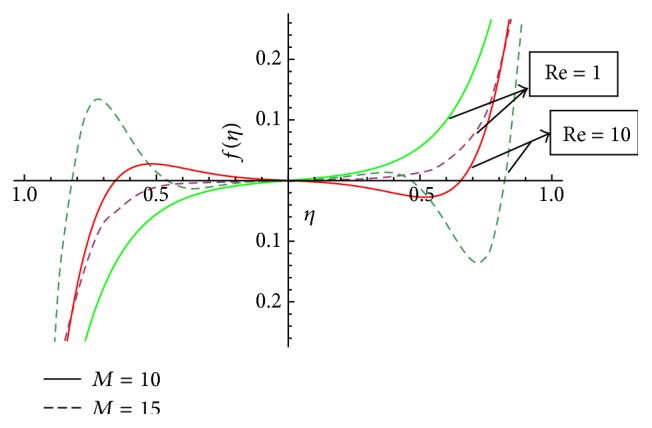
Variation of *f*(*η*) with the Reynolds number (Re) with *K* = 2.

**Figure 4:**
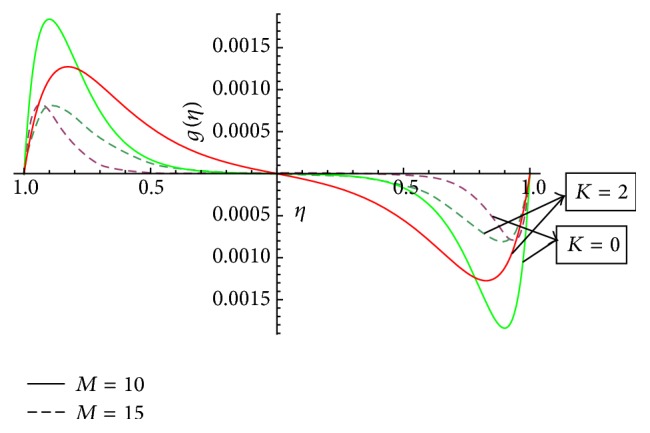
Variation of *g*(*η*) with the Hartmann number (*M*) and *K* = 2.

**Figure 5:**
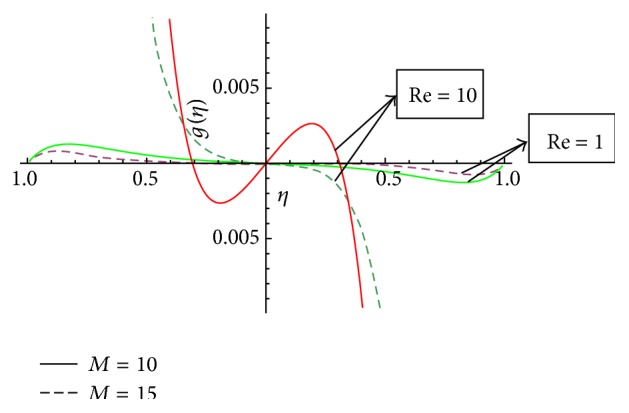
Variation of secondary velocity *g*(*η*) with the Reynolds number (Re) and *K* = 2.

**Figure 6:**
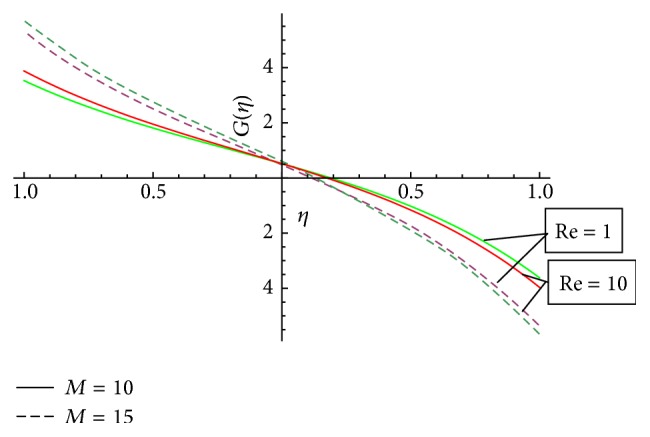
Variation of *G*(*η*) for the different values of the Reynolds number and the Hartmann number and *K* = 1.

**Figure 7:**
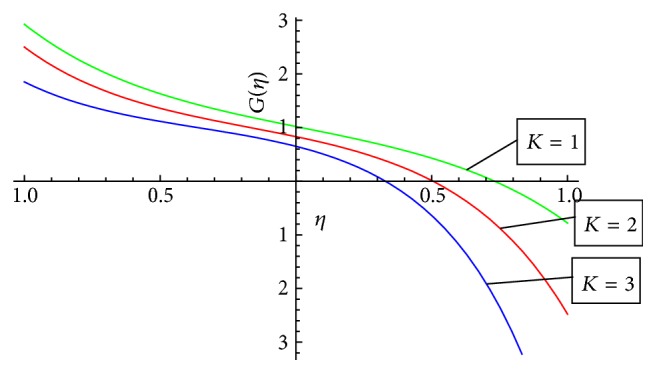
Variation of *G*(*η*) with micropolar parameter *K* for the Hartmann number *M* = 10 and the Reynolds number *Re* = 1.

In this section we have discussed the effect of vital parameters like the Hartmann numbers and the Reynolds numbers on the dimensionless velocities and the micromotion using Mathematica software.  Figure 2 explains the behavior of primary velocity *f*(*η*) with the Hartmann number and micropolar parameter. It is observed that, with the increase of the Hartmann number *M*, the primary velocity *f*(*η*) decreases towards the upper disk and increases towards the lower disk. This accelerating and retarding effect on primary velocity *f*(*η*) is due to the Lorentz force. It is noticed that the primary velocity in the core region is nearly uniform. It has a more pronounced variation near the disks. With the increase of micropolar parameter *K*, the primary velocity *f*(*η*) decreases towards the lower disk and increases towards the upper disk. It is noticed that the primary velocity in the core region is nearly uniform. It has pronounced variations near the disks representing that the micropolar parameter *K* has retarding influence on the magnitude of the primary velocity in the vicinity of the lower disk and accelerates the primary velocity near the upper disk.  Figure 3 represents the variation of primary velocity *f*(*η*) with the Reynolds number. It is observed that the primary velocity near to the disk on the right side of the axis decreases with the increase of the Reynolds number whereas the nature reverses on the left side of the axis. This claims the fact that Re is more influential in the vicinity of disks as compared to the core region. Near to the upper disk, for the higher value of the Reynolds number *Re* = 10, there is a flow reversal in the region 0.5 ≤ *η* ≤ 0.8 for the primary flow, whereas near to the lower disk, for the lower value of the Reynolds number *Re* = 1, there is a flow reversal in the region 0.5 ≤ *η* ≤ 0.8 for the primary flow. For the region −0.5 ≤ *η* ≤ 0.5, primary velocity *f*(*η*) appears to be uniform.  Figure 4 shows the influence of micropolar parameter *K* and a Hartmann number *M* on secondary velocity *g*(*η*). It is observed that the flow near to the disk on the right side of an axis increases with the increase of *K* and *M* whereas the nature of the secondary velocity reverses on the left side of the axis. With the increase of the Hartmann number *M*, flow in the core region appears to be more stagnant.  Figure 5 shows the variation of secondary velocity with the Reynolds number. It reveals that, with the increase of the Reynolds number, the secondary velocity increases. For the low Reynolds number *Re* = 1, there is a flow reversal in the region 0.2 ≤ *η* ≤ 1, and for the region 0.0 ≤ *η* ≤ 0.2, flow appears to be uniform.  Figure 6 shows the variation of micromotion of fluid with the Reynolds number (*Re*) and the Hartmann number (*M*). It has been observed that the micromotion of the fluid near to the disk on the right side of the axis increases with the decrease of the Reynolds number where as the nature of the micromotion of the fluid reverses on the left side of the axis. The behavior of the Reynolds number on the micromotion of the fluid is same as that of the Hartmann number (*M*).  Figure 7 represents the variation of micromotion of fluid with micropolar parameter. It has been observed from the Figure 7 that an increase in micropolar parameter *K* results in slowing down of micromotion of fluid.

## 4. Conclusion

This work investigates the flow of an incompressible micropolar fluid between eccentrically placed disks. An exact solution for the governing equations has been obtained in a closed form. It is found that the primary velocity has an accelerating and the retarding effect due to the Lorentz force. Micropolar parameter *K* has retarding influence on the magnitude of the primary velocity in the vicinity of the lower disk and accelerates the primary velocity near the upper disk. The Reynolds number is more influential in the vicinity of disks as compared to the core region. Flow reversal occurs for the lower value of the Reynolds number. The behavior of the Reynolds number on the micromotion of the fluid is same as that of the Hartmann number (*M*). It has been observed that an increase in micropolar parameter *K* results in slowing down of micromotion of fluid.

## Nomenclature

*K*: Micropolar parameter
Re: The Reynolds number
*M*: The Hartmann number
*α*, *β*: Gyroviscosities
*γ*: Spin-gradient viscosity
*κ*: Vortex viscosity
*μ*: Dynamic viscosity
*j*: Microinertia per unit mass.

## References

[1] Eringen A. C. (1966). Simple microfluids. *International Journal of Engineering Science*.

[2] Eringen A. C. (1966). Theory of micropolar fluids. *International Journal of Mathematics and Mechanics*.

[3] Eringen A. C. (1972). Theory of micropolar fluids. *Journal of Mathematical Analysis and Applications*.

[4] Tan W. C., Masuoka T. (2005). Stokes’ first problem for a second grade fluid in a porous half-space with heated boundary. *International Journal of Non-Linear Mechanics*.

[5] Tan W. C., Masuoka T. (2005). Stokes’ first problem for an Oldroyd-B fluid in a porous half space. *Physics of Fluids*.

[6] Fetecau C., Fetecau C. (2005). Decay of a potential vortex in an Oldroyd-B fluid. *International Journal of Engineering Science*.

[7] Fetecau C., Fetecau C. (2005). Unsteady flows of Oldroyd-B fluids in a channel of rectangular cross-section. *International Journal of Non-Linear Mechanics*.

[8] Fetecau C., Fetecau C. (2005). Starting solutions for some unsteady unidirectional flows of a second grade fluid. *International Journal of Engineering Sciences*.

[9] Hayat T., Nadeem S., Asghar S., Siddiqui A. M. (2006). Unsteady MHD flow due to
eccentrically rotating porous disk and a third grade fluid at infinity. *International Journal of Applied Mechanics and Engineering*.

[10] Hayat T., Kara A. H. (2006). Couette flow of a third-grade fluid with variable magnetic field. *Mathematical and Computer Modelling*.

[11] Hayat T., Khan S. B., Khan M. (2007). The influence of Hall current on the rotating oscillating flows of an Oldroyd-B fluid in a porous medium. *Nonlinear Dynamics*.

[12] Chen C.-I., Chen C.-K., Yang Y.-T. (2004). Unsteady unidirectional flow of an Oldroyd-B fluid in a circular duct with different given volume flow rate conditions. *Heat and Mass Transfer*.

[13] Chen C.-I., Chen C.-K., Yang Y.-T. (2003). Unsteady unidirectional flow of second grade fluid between the parallel plates with different given volume flow rate conditions. *Applied Mathematics and Computation*.

[14] Berker R. (1963). *Integration des Equations du Movement d’un Fluid Visquent Incompressible, Hand Book of Fluid Dynamics*.

[15] Coirier J. (1972). Rotations non coaxials d’un disque et d’un fluide a I’infini. *Journal de Mecanique*.

[16] Abbott T. N. G., Walters K. (1970). Theory for the orthogonal rheometer, including an exact solution of the Navier-Stokes equations. *Journal of Fluid Mechanics*.

[17] Mohanty H. K. (1972). Hydromagnetic flow between two rotating disks with noncoincident parallel axes of rotation. *Physics of Fluids*.

[18] Rao A. R., Kasiviswanathan S. R. (1987). A class of exact solutions for the flow of a micropolar fluid. *International Journal of Engineering Science*.

[19] Rajagopal K. R. (1996). On an exact solution for the flow of an Oldroyd-B fluid. *Bulletin of the Technical University of Istanbul*.

[20] Ersoy H. V. (1999). MHD flow of an Oldroyd-B fluid between eccentric rotating disks. *International Journal of Engineering Science*.

[21] Erdogan M. E. (1995). Unsteady viscous flow between eccentric rotating disks. *International Journal of Non-Linear Mechanics*.

[22] Rao A. R., Kasiviswanathan S. R. (1987). On exact solutions of the unsteady Navier-Stokes equations-the vortex with instantaneous curvilinear axis. *International Journal of Engineering Science*.

[23] Lai C.-Y., Rajagopal K. R., Szeri A. Z. (1984). Asymmetric flow between parallel rotating disks. *Journal of Fluid Mechanics*.

[24] Knight D. G. (1980). Flow between eccentric disks rotating at different speeds: inertia effects. *Zeitschrift für Angewandte Mathematik und Physik*.

[25] Guria M., Jana R. N., Ghosh S. K. (2007). Unsteady MHD flow between two disks with non-coincident parallel axes of rotation. *International Journal of Fluid Mechanics Research*.

[26] Guria M., Das B. K., Jana R. N. (2007). Oscillatory flow due to eccentrically rotating porous disk and a fluid at infinity. *Meccanica*.

[27] Guria M., Das B. K., Jana R. N. (2007). Hydromagnetic flow due to eccentrically rotating porous disk and a fluid at infinity. *International Journal of Fluid Mechanics Research*.

[28] Guria M., Das S., Jana R. N. (2007). Hall effects on unsteady flow of a viscous fluid due to non-coaxial rotation of a porous disk and a fluid at infinity. *International Journal of Non-Linear Mechanics*.

[29] Shercliff J. A. (1965). *A Textbook of Magnetohydrodynamics*.

[30] Capriz G. (1989). *Continua with Microstructures, Springer Tracts in Natural Philosophy*.

[31] Mariano P. M. (2005). Migration of substructures in complex fluids. *Journal of Physics A: Mathematical and General*.

